# The influence of Arctic amplification on mid-latitude summer circulation

**DOI:** 10.1038/s41467-018-05256-8

**Published:** 2018-08-20

**Authors:** D. Coumou, G. Di Capua, S. Vavrus, L. Wang, S. Wang

**Affiliations:** 1 Department of Water & Climate Risk, Institute for Environmental Studies, VU Amsterdam, Amsterdam, 1087HV Netherlands; 2 Department of Earth System Analyses, Potsdam Institute for Climate Impact Research, Potsdam, 14473 Germany; 3 Nelson Institute Center for Climatic Research, University of Wisconsin-Madison, Madison, 53706 WI USA; 4 Department of Earth and Planetary Sciences, Harvard University, Cambridge, 02138 MA USA; 5 Department of Plants, Soils and Climate, Utah State University, Logan, 84322 UT USA

## Abstract

Accelerated warming in the Arctic, as compared to the rest of the globe, might have profound impacts on mid-latitude weather. Most studies analyzing Arctic links to mid-latitude weather focused on winter, yet recent summers have seen strong reductions in sea-ice extent and snow cover, a weakened equator-to-pole thermal gradient and associated weakening of the mid-latitude circulation. We review the scientific evidence behind three leading hypotheses on the influence of Arctic changes on mid-latitude summer weather: Weakened storm tracks, shifted jet streams, and amplified quasi-stationary waves. We show that interactions between Arctic teleconnections and other remote and regional feedback processes could lead to more persistent hot-dry extremes in the mid-latitudes. The exact nature of these non-linear interactions is not well quantified but they provide potential high-impact risks for society.

## Abstract (web summary)

Accelerated global warming in the Arctic might have profound impacts on mid-latitude weather particularly in winter, although the evidence for an effect also in summer is also growing. Here Coumou et al. show that these interactions could lead to more persistent hot-dry extremes in mid-latitudes.

## Introduction

The observed increases in the frequency and intensity of extreme heat and heavy rainfall events since the late 1980s, especially in mid-latitude regions, have been linked to anthropogenic global warming^1–3^. Scientists are generally confident in the thermodynamic drivers of these changes but are less so in dynamic aspects^4,5^. Another pronounced signal of anthropogenic global warming is the rapidly increasing near-surface temperatures in the Arctic at a pace two to four times faster than the rest of the globe, known as Arctic amplification (AA)^6^. The extent to which AA affects the mid-latitude circulation and possibly contribute to the observed increases in weather extremes has been a subject of active debate^7^.

Most studies analyzing the role of AA on mid-latitude weather have focused on the winter season and the linkage with cold spells. The stronger jet stream, the presence of the stratospheric polar vortex, and the post-1990s increase in abnormally cold winters over central Eurasia have drawn a lot of attention to the winter season^6,8,9^. The increased heat stored in the Arctic Ocean owing to sea-ice loss is released into the atmosphere in early winter. The associated expansion of the near-surface air increases Arctic geopotential heights and can affect the circumglobal circulation directly as well as via feedbacks between the troposphere and stratosphere involving the stratospheric polar vortex^6,10–14^. Even though the exact pathways through which the Arctic influences the mid-latitude winter circulation are debated, a scientific consensus is emerging that AA has at least some influence on winter weather^7,15,16^.

**Fig. 1:**
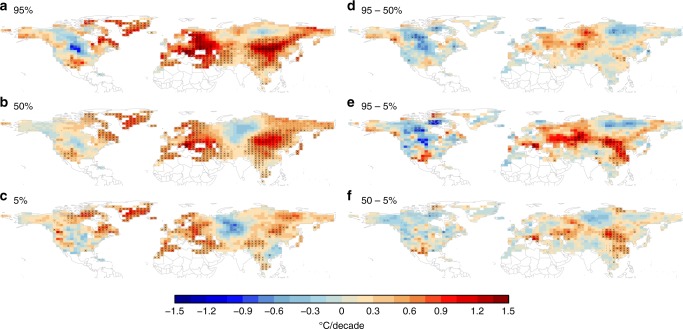
Summer trends in surface temperature over 1980–2011. **a** 95th, **b** 50th, and **c** 5th quantile of the HadGHCND^133^ gridded daily dataset; differences in the trends of different quantiles, plotted in **d**–**f**, reflect changes in the width of the distribution. Over most mid-latitude regions, especially over Eurasia, the width of the distribution has broadened and thus variability has increased. (Figure created using R statistical software)

Links between AA and summer circulation have received far less scientific attention, despite the potential for synergistic effects that might favor high-impact extremes. In summer, thermodynamic and dynamic drivers of extreme weather could act in the same direction, leading to tail risks^17^. For instance, any increased frequency in circulation regimes conducive to persistent heat extremes would act on top of the thermodynamically driven increase in heat, creating possibilities for very-extreme heatwaves. Many recent high-impact summer heatwaves indeed occurred in that far-tail of the distribution and cannot be explained by the direct thermodynamic effect of greenhouse gas forcing alone (Box 1)^18^^–^^21^. Such extreme heatwaves have been found to increase and intensify across most regions but more so in the mid-latitudes than over the rest of the globe^20^. Consistent with the increase in heatwaves, the hot tail of summer temperature distribution has been warming faster than the median and the cold tail. Figure 1 shows the warming trends in the 95th percentile (hot tail), 50th percentile (median), and 5th percentile (cold tail) of daily summer temperatures. Clearly, over most mid-latitude regions, in particular over Eurasia but less so in the US, the hot tail has been warming faster than the cold tail and thus temperature variability in summer has increased^22^. This increased variability indicates that more complex processes beyond simple radiative greenhouse gas forcing are important in driving heat extremes (Box 1). This is supported by recent studies that indicate that summer weather has become more persistent in several regions in the mid-latitudes^23–25^. In summer, the hot tail of the distribution is associated with persistent, blocking weather systems, and an increase in their persistence leads to more extreme temperatures.

Here, we review recent studies analyzing possible links between AA, mid-latitude summer weather and extreme events, in particular persistent hot-dry extremes. We start by giving a brief synopsis of the different Arctic mechanisms proposed for winter. Next, we address the seasonal differences in the mid-latitude circulations, in the influence of regional and far-away drivers, and in detected changes in the Arctic. We focus on three possible dynamical pathways that are most relevant to summer and summarize the theoretical, empirical, and modeling evidence for each of them. We discuss the confidence and uncertainties associated with these dynamical pathways, identify knowledge gaps and key societal risks, and provide a roadmap for future research.

### BOX 1 Recent mid-latitude summer weather extremes and their impacts

Many recent high-impact summer heatwaves occurred in the far-tail of the distribution and are difficult to explain by the direct radiative warming effect of greenhouse gas forcing alone^18–21^. In 2010, Russia saw 33 consecutive hot-and-dry days (with temperatures above 30 °C), resulting in an estimated 55,000 heat-related deaths, more than 500 wildfires near Moscow and grain-harvest losses of 30%^4^. A quantitative global analyses showed that the 2010 event was the most-severe heatwave ever recorded worldwide, based on a heatwave index that can be used across different regions^20^. Intriguingly, all record-setting heatwaves based on this index occurred in the mid-latitudes, indicating that here heatwaves are becoming more intense at a pace that exceeds the global mean^20^. Extreme summer heat in the Northern Hemisphere mid-latitudes now far exceeds historical frequencies in the twentieth century^117^. Over the last decade, Europe has seen an exceptionally rapid increase in the chance of extremely hot summers similar to the 2003 extreme^21^. Other notable high-impact and record-breaking droughts and heatwaves occurred in the USA in 2011 and 2012^4,20^, leading to billions of dollars in agricultural losses^120,121^.

One reason why these extremes cause so much damage is that temperature and precipitation during summer are anti-correlated virtually everywhere across extratropical land^122^. Consequently, extremes of heat and dryness often coincide to produce compound extremes that exert disproportionately large societal impacts^123^. Certain mid-latitude regions identified as hotspots of tight atmosphere–land coupling, such as central North America ^124^, are especially prone to concurrent heatwaves and droughts.

Such events are promoted by positive thermodynamic feedbacks that favor depleted soil moisture and enhanced sensible surface heating as the land warms, but they are also strongly regulated by atmospheric dynamics that initiate and sustain anomalous heating and drying^125^. These kinds of summertime compound events are therefore highly relevant for the dynamical changes described in this study, and recent studies have developed innovative techniques to separate the contributions from dynamics and thermodynamics^126–128^. In particular, the projected trend toward a weaker and poleward-shifted jet stream is consistent with projections of a significantly increased risk of compound hot-dry extremes across much of the Northern Hemisphere this century^122^. This type of climate change would likely exacerbate the separate impacts of extreme heat and dryness, based on the documented stresses that compound heatwaves and droughts exert in causing disease^129^, vegetation mortality^130^, wildfires^131^, and agricultural losses^132^.

## Arctic amplification and mid-latitude winter circulation

Due to declining sea-ice, the Arctic Ocean absorbs more incoming solar radiation from spring to autumn. By early winter, when near-surface air temperatures drop below sea-surface temperatures, this excessive heat is released into the atmosphere^6,10–13^. The additional heat inflates the lower troposphere over the Arctic Ocean and nearby continents, and increase geopotential heights, which could affect circulation patterns further south.

**Fig. 2:**
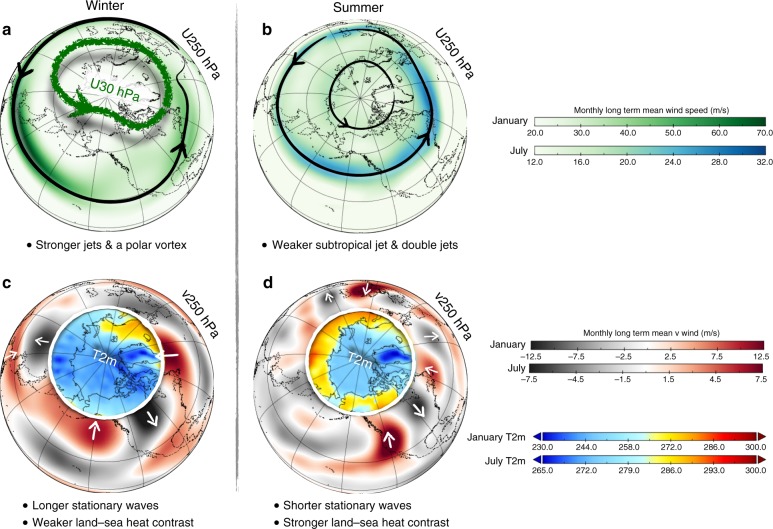
Schematic figure illustrating the main seasonal differences in upper tropospheric circulation between winter (January) and summer (July). Panels **a** and **b** show 250-hPa wind speed (green-to-blue shading) illustrating the jet streams with black arrow lines that follow the zone of maximum wind speed. The wintertime stratospheric polar vortex is outlined with the thick green line following the 30-hPa maximum wind speed. Panels **c** and **d** show the 250 hPa meridional wind speed (dark gray-to-dark red shading) depicting the stationary wave features associated with the jet streams. White arrows are added to illustrate wind direction. Basic differences in the summer circulation features, as compared to winter, include shorter stationary waves, more northerly subtropical jet, absence of stratospheric polar vortex and an Arctic front jet forming double jets. Data are 1970–2000 climatology of NCEP Reanalysis^134^ (downloadable: https://www.esrl.noaa.gov/psd/data/gridded/data.ncep.reanalysis.html). (Figure created using Panoply and Apple’s Keynote software)

The observed increase in Arctic geopotential heights^6,14^ might reduce the poleward pressure gradient in the troposphere and therefore weaken the storm tracks and westerly jet. However, this notion based on thermal-wind balance and baroclinicity provides little explanation for the recent changes in winter circulation. In winter, the near-surface warming in the Arctic has been pronounced but confined to high latitudes only, i.e., north of 70° N^6^. Within the mid-latitudes (i.e., 30° N–60° N), neither the poleward temperature gradient nor the zonal-mean jet or storm track have seen any significant changes in winter^26^. Future climate model projections under high-emission scenarios show that both changes in the tropics^27,28^ and in the Arctic^29–31^ can influence the strength and position of the mid-latitude winter circulation. Enhanced warming projected in the tropical tropopause region (due to enhanced deep convection and latent heating) acts to increase the upper-level poleward temperature gradient, which strengthens the mid-latitude westerlies^28^. This has become known as the tug-of-war (Fig. 2), whereby tropical changes tend to strengthen mid-latitude circulation and lead to a poleward migration, whereas AA has the opposite effect^32^.

Many recent winters were characterized by extremely warm temperatures in the Arctic and anomalously cold conditions further south, especially over Eurasia. In fact, large areas over central Eurasia have been cooling since 1990^9^. Possible dynamical mechanisms behind this warm-Arctic cold-continent pattern involving sea-ice loss consist of a direct tropospheric pathway^33^ and pathways involving the stratospheric polar vortex (Fig. 2)^34^. In the latter hypothesis, increased geopotential heights over high-latitude regions can cause a pronounced upward wave propagation into the stratosphere which can weaken the stratospheric polar vortex and, in extreme cases, trigger sudden stratospheric warming events. A weak polar vortex can propagate downwards into the troposphere causing a negative Arctic oscillation (AO) that is conducive to cold spells in Eurasia and Siberia^34^.

There is relatively high confidence that this stratospheric pathway is a real phenomenon and has a role in the warm-Arctic cold-continent pattern, as it is supported by multiple lines of evidence: From empirical analyses, to causal discovery algorithms, and climate model simulations^6,15,35,36^. Still, its relative importance compared to other pathways, tropical influences and atmosphere internal variability remains unclear^7^.

## Summer circulation

Compared to winter, summer circulation in the mid-latitude is weaker, more barotropic and the climatological jets are more zonally oriented, which promotes the formation of circumglobal wave trains (CGWT, see Box 2). It is less influenced by variability in tropical sea-surface temperatures (SST), and more sensitive to land-atmosphere feedbacks involving soil moisture or snow cover.

In summer, the reduced pole-to-equator temperature gradient (as compared to winter) leads to weaker and more-narrow upper-level westerlies, and the stratospheric polar vortex is absent all together (Fig. 2). The role of the stratosphere in influencing boreal summer weather is therefore considered non-existent^37^. Moreover, variations in tropical SST have less influence on mid-latitude circulation in summer compared to winter (e.g., refs.^38,^^39^). The position of abnormally warm SST in the tropics determines where the strongest deep convection takes place associated with shifts in the Walker circulation. The upper-level latent heat release during deep convection can trigger long Rossby waves that propagate poleward and influence mid-latitude weather^40^. This mechanism is less important in summer than winter for two reasons: First, the El Niño-Southern Oscillation (ENSO), the dominant mode of variability in tropical SST, tends to peak in boreal winter and is much weaker during boreal summer^41^. Second, the prevailing easterly winds in the tropics in summer limit the ability of Rossby waves to propagate poleward^38^. This is not to say that the tropics cannot influence summer mid-latitude weather. Among other things, ENSO gives some predictive seasonal forecast skill in summer^42,43^ and the varying location and intensity of monsoon systems, notably over South and East Asia, can affect the mid-latitude summer circulation^44–46^.

The state of the cryosphere, in terms of sea-ice and snow cover, from late winter to early summer can influence the strength and latitude of the summer time jet^47^. Boreal snow cover during spring and summer has shrunk dramatically in recent years, even faster than the decline of Arctic sea-ice extent^48,49^. Without snow, the surface albedo is lower and thus the land regions absorb more incoming solar radiation. Furthermore, declining snow cover in spring has a delayed drying effect on the soils by mid-summer, favoring enhanced temperatures due to suppressed evaporative cooling^50,51^. These thermodynamic processes in conjunction with reductions in early season snow cover can affect regional to hemispheric circulation^52,53^.

The waveguide effect, i.e., the trapping and focusing effects of the seasonal jet streams on low-frequency tropospheric waves, has an important role in how a changing mid-latitude circulation might promote stagnant weather patterns^39,54^. Waveguides produce zonally oriented chains of perturbations that fluctuate at relatively low frequency ranging from weeks to months, creating a teleconnection pattern. Early research found that atmospheric disturbances near the jet core are refracted toward the core, meaning that the jet acts as a waveguide^55^. The energy of the trapped disturbances does not disperse strongly and therefore can propagate much further and possibly become circumglobal. Such CGWT can generate heatwaves and severe weather outbreaks (e.g., flooding) due to their longer lifetime than synoptic disturbances^56,57^.

**Fig. 3:**
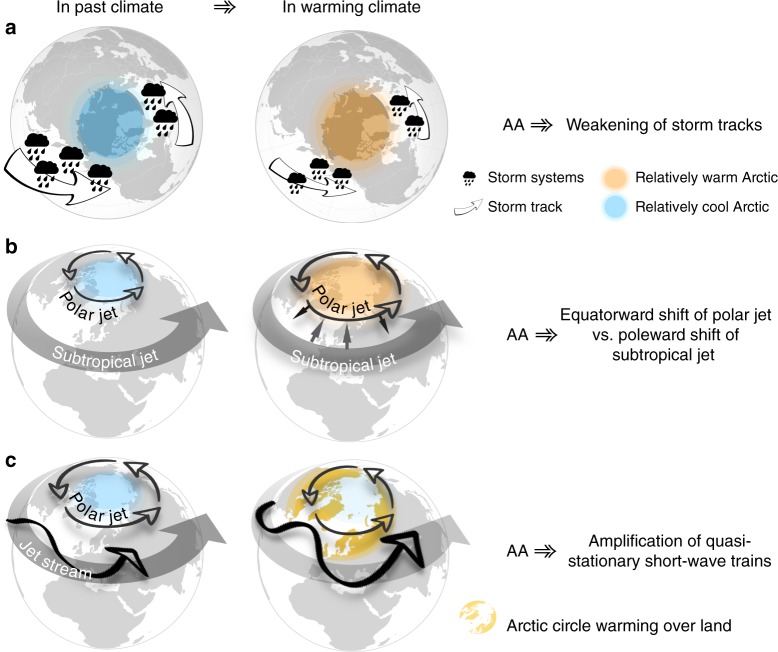
Schematic representation of proposed dynamical mechanisms in summer. **a** Weakening of storm tracks, **b** latitudinal-shift in jet positions, and **c** amplification of quasi-stationary waves (Figure created using Apple’s Keynote software)

The climatological jets in summer have less northward tilt compared to winter and therefore waveguides are also oriented west-to-east and have the potential to become circumglobal (Fig. 2). Also, the narrower mean jet in summer favors the waveguide effect with wave trains orienting zonally along the jet stream waveguide^38,56,58,59^. However, the zonal orientation of the climatological jet does not mean that the overall flow is less wavy in summer compared to winter: The total waviness in geopotential height fields on sub-synoptic to sub-seasonal time scales is as pronounced in summer as in winter, if not more^60–62^. In winter, Rossby waves are typically oriented along a meridional path arcing from the tropics into the mid-latitudes, or from the mid-latitudes into the tropics (see Fig. 3 of Hoskins and Woollings^63^). Due to the zonal orientation in summer, any local heating anomaly can generate a sequence of waves of similar wavelength downstream of the jet, forming a stagnant wave packet that affects weather conditions far away. Recent research has shown that when synoptic-scale waves (wavenumbers 6–8) are trapped in a (near) circumglobal waveguide, wave-resonance can greatly increase their amplitude^19,64–66^. The wave-resonance mechanism can lead to highly persistent and anomalous weather conditions around the hemisphere and studies have linked it to several recent high-impact summer extremes, including heatwaves and floods^57,66^. Though there is a solid theoretical basis underlying wave-resonances, their exact significance in the real-world in causing extreme weather events is debated^17^.

Finally, the Arctic frontal zone that develops around 70° N in summer^67^ is likely to be affected by AA. The warm, snow-free land surface and the cold Arctic Ocean create a strong thermal contrast along the Arctic seaboard around 70° N, generating strong westerlies here. These sub-polar westerlies, together with the pronounced sub-tropical jet, form the distinct summer feature of double jets (Fig. 2). A double-jet regime is characterized by a very confined sub-tropical jet with sharp edges wherein wind speeds change rapidly with latitude. Such sharp sub-tropical jets are effective waveguides^68^ and thus double jets favor waveguide formation and wave-resonance events^69^. Interactions of the two jets can produce high-amplitude atmospheric waves, creating the deepening of troughs and stagnation of ridges^66,70^.

Given these specific characteristics of the summer circulations, several mechanisms have been proposed that link AA with summer mid-latitude weather patterns. These are grouped into weakening of the storm tracks, shift in the latitudinal position of the mid-latitude jet, and amplification of circumglobal wave trains (Fig. 3).

## Influence of the Arctic on summer circulation

### Weakening storm tracks

Theoretical, observational and modeling evidence supports the hypothesis that summer storm tracks weaken with enhanced Arctic warming^26,71,72^. The theoretical basis underlying AA and resultant weakening of the mid-latitude storm track is straightforward: The thermal-wind balance relates vertical shear in the westerly flow to the magnitude of the poleward temperature gradient. In the lower troposphere, a reduction in the temperature gradient equates to a similar reduction in the shear, weakening the thermally driven jet and reducing the low-level baroclinicity^63^. A reduced low-level baroclinicity implies less or weaker synoptic-scale cyclogenesis and thus leads to overall weakening of the storm tracks. Note, that the thermal-wind balance does not give a direction of causality per se: The causality could be the other way around, whereby a change in mid-latitude circulation alters the poleward heat transport giving rise to more rapid warming in the Arctic^73^.

Empirical evidence based on multiple datasets shows that over the satellite-covered period (i.e., since 1979), the mid-latitude summer circulation has indeed weakened in conjunction with a reduction in the poleward temperature gradient in the lower troposphere. This weakening has been detected in the westerly jet (following the thermal-wind balance), the total kinetic energy of synoptic storm systems (by about 15%) and the number of strong cyclones^26,71,74^. Similarly, strong Arctic sea-ice melting years are characterized by a weakened circulation^75^. While, the satellite era is most reliable when analyzing wind field characteristics, its limited timespan compromises long-term trend analyses. Natural variability on multi-decadal time scales, either due to changes in SSTs or from internal atmospheric variability, are thus likely to have a role in the observed trends.

There is modeling evidence indicating that these observed trends are at least partly attributable to AA. CMIP5 coupled model simulations of the twentieth century show that the observed changes in the zonal-mean temperature gradient in summer (characterized by AA and enhanced high-latitude land warming) are likely attributable to anthropogenic forcing ("likely" according to IPCC lexicon)^76^. Idealized modeling experiments support storm track weakening when sea-ice is reduced but also indicate that sea-ice changes by itself can explain only part of the observed weakening^77^. Modeling studies indicate that the effects of historic sea-ice reductions can explain up to one-third of the magnitude of the observed anomalies, with an additional role for changes in SSTs^72,78^. Thus, other factors including natural variability likely had a role in the recently observed summer circulation changes, but a substantial share of it is likely attributable to AA.

**Fig. 4:**
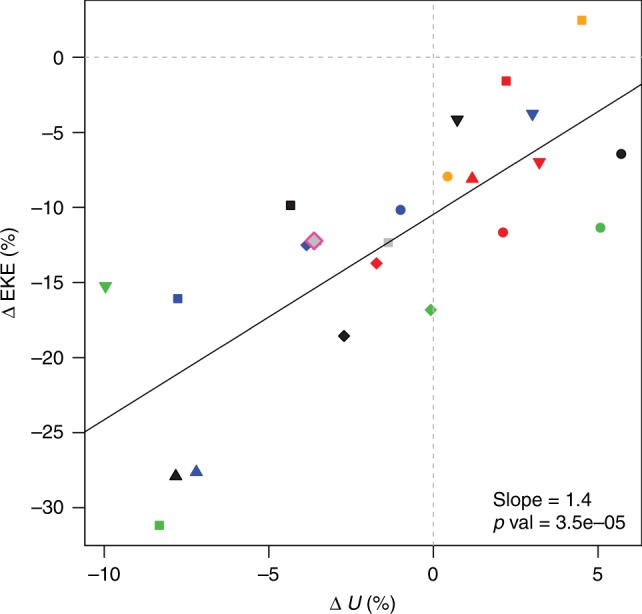
Observed and projected changes in the mid-latitude Northern Hemisphere summer storm tracks and westerlies. The percentage change in summer storm tracks (vertical axis) and westerlies (horizontal axis) in future (2081–2100, under scenario RCP8.5) relative to 1981–2000 for individual CMIP5 climate models is shown, and their linear fit (solid black line). Observed changes based on ERA-Interim data are given for the 1979–2013 period. Taken from (Coumou et al.^26^.)

For future high-emission scenarios, models robustly project storm track weakening, supporting the hypothesis that AA is associated with weakened summer storm tracks (see Fig. 4)^26,71,79,80^. The changes at the end of the century in the high-emission scenario are comparable to the observed changes over the past decades^26^. This suggests that either the models underestimate the future changes (models also underestimate historic changes in the Arctic itself) or that a substantial part of the observed trend is associated with multi-decadal natural variability^81^. For the North American sector, the amount of AA in models by the end of century is negatively correlated with the changes in jet speed and wave phase speed in summer^28^. Models robustly project a weakening of summer storm tracks by the end of the century (Fig. 4), but this is not the case for the upper-level jet: There is large inter-model spread with some models projecting a strengthening and some a weakening of the upper-level jet (Fig. 4)^26,28^.

### Shift in jet-position

Understanding jet shifts in the Northern Hemisphere is a challenging task due to several competing processes. Theoretically, the change in jet stream position can be divided into a relatively small direct radiative-induced shift and a larger indirect SST-mediated effect. The indirect effect comes from mid-latitude dynamical feedbacks, involving enhanced irreversible mixing due to wave breaking of high-frequency transient eddies^82^, and can explain most of the expected jet shifts^83^. When considered in isolation, AA should theoretically cause a southward shift in the mid-latitude jet stream.

Idealized dry atmospheric model simulations indeed indicate a southward shifted jet coming from AA by itself^84^. This is also confirmed by more complex models with reductions in sea-ice imposed^31,72,78,85^. Despite the process linking AA to a more equatorward jet, the zonal-mean jet streams are projected to migrate poleward by about one degree by the end of the twenty-first century under a high-emission scenario^27,86^. Thus in the long run, i.e., at the end-of-century, the tropics likely dominate the tug-of-war, at least in models. While in winter both the Atlantic and the Pacific jets are projected to migrate poleward, in summer this shift is seen only for the North Atlantic jet^28^. AA may therefore exert a stronger opposing influence to the expected poleward shift of the Pacific jet in summer. Different state-of-the-art climate models employ different simplifying parameterizations (e.g., for clouds) and those can, in a complex, non-linear system, lead to very different outcomes^87^. The inter-model spread of the poleward shift therefore tends to be larger than the signal itself^27,46,86^.

Observed jet shifts in the Northern Hemisphere are generally small compared to those in the Southern Hemisphere, but still generally indicate a poleward migration^88^. For the Northern Hemisphere, some evidence of a poleward shift of jet streams has been identified in reanalysis products^88,89^ and satellite observations of clouds^90,91^ and is most significant in winter. This is thus largely in agreement with model projections pointing at the important role of the tropics in shifts in the jet position.

### Amplification of wave trains

**Fig. 5:**
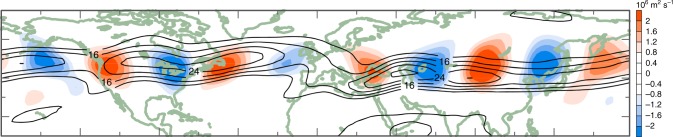
Enhanced circumglobal wave train embedded in the summer jet. Linear trends from 1979 to 2010 in the July 250 hPa stream function in the short-wave regime (blue-red shading) computed with the long wavenumbers (1–4) removed. The change in the short waves is embedded in the climatological July-mean 250-hPa wind speed depicting the jet stream (black contour lines). Adapted from (Wang et al.^95^.)

Limited evidence from theory, observations and some model simulations suggests that AA may amplify synoptic-scale, quasi-stationary waves embedded in the summer jet (Fig. 5). Theory of the dynamics of a dry atmosphere suggests that a lower troposphere diabatic heating source in the mid-latitudes will have a larger stationary wave response (in terms of a meridional stream function displacement) when the background baroclinicity and zonal winds are reduced, as a direct response of AA^63^. In a more complex atmosphere of an aquaplanet model (i.e., an Earth covered by water only), quasi-stationary synoptic-scale wave trains are enhanced when the meridional temperature gradient is reduced and the westerly winds weaken^92^. Thus, as the background flow becomes weaker, the same heating source in the lower troposphere can trigger a stronger stationary wave response especially for synoptic-scale waves. The increased moisture content in a warmer atmosphere, and the tendency for increased latent heat release in the tropics and over warm ocean currents in higher latitudes, can provide further heating to perturb more or stronger CGWTs.

Some observational evidence suggests that the quasi-stationary component of mid-latitude summer circulation has become wavier since 1979, in particular over the North American sector^60,62,93,94^. Figure 5 plots linear trends over 1979–2010 in the short-wave regime showing enhanced CGWTs over both the North American and Eurasian sectors. For the American sector, this is further supported by detected increases in waviness metrics^60,62^ (see Box 3). Using three different climate models, the summer time amplification of quasi-stationary short waves over the American sector appears to be attributable to greenhouse gas forcing^95^. As the CGWT is linked to the summer North Atlantic Oscillation (SNAO, Box 2)^39^, an AA-like circulation anomaly (i.e., negative phase of the SNAO) could modulate the CGWT and vice versa. However, the summer CGWT can also be triggered by heating sources associated with the Indian monsoon^44^.

The few modeling studies with historically observed low sea-ice concentration show a stationary wave-train response in summer emerging from AA^78,95^. Still, future model projections of mid-latitude quasi-stationary short-wave patterns are generally inconsistent with recent observations. Future CMIP5 projections under high-emission scenarios show an overall decrease in blocking both for winter and summer^96^. The reasons behind these divergent findings are not well understood and may result from competing effects from the tropical monsoons^44^, changes in land-sea thermal contrast^46^, model biases in representing summer time Rossby waves^39^ and the use of different diagnostics to quantify waviness (see Box 3).

### BOX 2 Atmospheric teleconnections in summer

Remote climate effects, known as atmospheric teleconnections, consist of three main types: (i) regional long-wave pattern confined in a longitudinal sector of the globe, like the Pacific-North America pattern and the North Atlantic Oscillation^135^; (ii) a hemisphere-wide pattern with a prominent zonal-mean component like the Arctic Oscillation or the Annular Modes^70,136,137^; and (iii) the trapping and focusing effects of the seasonal jet streams on low-frequency tropospheric waves^39^, known as the waveguide effect^54^. The figure below shows schematic representations of these three types of teleconnection patterns.

The summer North Atlantic Oscillation (SNAO) can be regarded as the counterpart of the more robust winter NAO. The centers of action of the SNAO exhibit a more northerly location, have a smaller geographical extent and a weaker dipole pattern compared to the winter NAO. Panel a schematically shows the surface pressure patterns of the positive SNAO phase, i.e., when pressure differences are strong, together with the position of the warmer/drier and colder/wetter regions. Generally, the negative phase shows a reverse pattern. Like the winter NAO, the impact of the SNAO on climate extremes such as heavy rainfall and flooding is profound, especially for Europe. Post-2007 summers have seen increasingly robust negative SNAO associated with a persistent anticyclonic anomaly over Greenland and a cyclonic anomaly over Northwest Europe. This pattern caused rapid melting of the Greenland ice sheet and brought unusually wet summers to Northwest Europe, including the massive flooding of U.K. in summer 2012. Future projections of climate models suggest an increasingly positive SNAO in warmer climates^138^.

The Northern Hemisphere Annular Mode (NAM) is an internally driven atmospheric mode maintained by both stationary and transient waves. The NAM is defined as the first EOF of monthly 500 hPa geopotential height fields and coincides with the definition of the Arctic Oscillation during the winter months^137^. Winter and summer NAM patterns present both a diverse geopotential height field, mean meridional circulation and eddy structure. The summer NAM has a smaller latitudinal extent and has a stronger link to surface air temperature over Eurasia. The positive phase of the summer NAM is associated with negative geopotential height anomalies over Greenland and the Arctic Ocean and an annual band of positive anomalies comprised between 40° and 60° N and particularly extended over Eurasia, as schematically shown in panel b. When the summer NAM is strongly positive, the storm tracks follow the Arctic front^137^. Anomalously positive summer NAM phases are associated with double jets favoring blocking between the polar and the subtropical jets. During a positive summer NAM phase, surface temperatures over Eurasia show a dipole pattern with warmer conditions over Europe and colder conditions over East Asia^70^.

The energy of waves trapped in a waveguide is not dispersed as broadly as in teleconnections of type (i) and therefore it can propagate farther before being dissipated. In summer, when an efficiently trapping waveguide becomes (almost) circumglobal, then resonant interactions between free and forced waves (typically of synoptic scale, wavenumbers 6–8) might lead to wave-amplification and persistent, high-amplitude waves. Waveguide formation is favored during double-jet regimes and thus linked to strongly positive NAM phases. Panel c shows a schematic representation of a wave-resonance event with an amplified wave 7. The orange and green areas represent regions of positive and negative upper-level meridional winds. Such events are associated with alternating hot-dry and cold-wet conditions, following the ridges and troughs. Such a situation is prone to blocking weather systems and deepened troughs, and their relative longevity is key to making severe weather extremes^19^. Wave-resonance periods have therefore been linked to both persistent heatwaves and severe flooding events^57,64^.

Finally, blocking itself is also distinctly different in summer compared to winter. Upstream latent heat release has been identified as an important contributor to persistent blocking and this mechanism is especially important in summer^139^.

**Figure Box 2:** Panel **a** shows a schematic of the positive SNAO indicating the anomalously low (blue) and high (red) sea level pressure regions together with cold/wet and dry/warm regions. Panel **b** shows a schematic of the 500 hPa geopotential height configuration during the positive phase of the NAM. Rainy clouds mark the position of the storm tracks during a strongly positive NAM phase. Panel **c** shows a schematic of an amplified wave 7. Green and orange regions show the position of pronounced northward and southward wind anomalies. (Figure created using Python, GIMP, Powerpoint and Inkscape software)

### BOX 3: Measuring mid-latitude waviness

Although the concept of wavy versus zonal circulation patterns is straightforward, quantifying these opposite states has proven challenging. Approaches can be roughly separated into geometric and dynamic methods. The former focuses on the geometry of the circulation to characterize the departure of the flow from zonality in terms of wave amplitude, sinuosity, or circularity^61,62,140,141^. These metrics have the advantage of being intuitive and readily visualized from geopotential height contours, but they have been criticized for lacking a firm physical basis. The existence of wave trains usually lasts 2–4 weeks and they do not necessarily have a preferred phase position^38^. Averaging over time may thus cancel out any signal. Further, often the diagnostic approach to detect extratropical jet’s waviness involves Fourier decomposition of zonal wavenumbers^58,64,65^ which could lead to spurious wave signals. In the extreme case: Fourier decomposition of a delta function will create amplitudes in a range of zonal wavenumbers even if the circulation feature is extremely local, i.e., it does not involve a wave “train”^63^. Likewise, a zonal wavenumber 5 along 40° N (subtropical jet) or 65° N (polar jet) depicts two very different wavelengths. Dynamically based waviness metrics such as effective diffusivity of potential vorticity^142^ and finite-amplitude wave activity^143,144^ are derived from first-order mass and circulation conservation principles and satisfy exact budget closure equations. These measures provide a theoretical basis for quantitatively relating changes in zonal wind speed to accompanying changes in wave amplitude and eddy fluxes, which is straightforwardly verifiable under idealized conditions. Such approaches are being applied in climatological studies of circulation trends and extreme weather events related to amplified flow patterns but their derivation is more technical and their application is more involved than geometric methods.

## Double jets

Basic theory suggests that double-jet flow regimes are to become slightly more common with pronounced AA^63^. Such flow regimes are characterized by sharper sub-tropical jets (i.e., a strong meridional wind shear) which can act as waveguides^68,69^. Moreover, in summer, recent AA is characterized by enhanced land warming over the high latitudes (~70° N) and much less warming over the nearby Arctic Ocean, in stark contrast to winter warming patterns. This enhanced high-latitude land warming is likely related to a combination of the smaller heat capacity of land compared to ocean, as well as late-spring to early-summer reduction of snow cover. Thus, while the overall equator-to-pole temperature gradient reduces, the thermal gradient actually increases at the land-ocean boundary around the Arctic circle. This situation favors the formation of the Arctic front jet at ~70° N in addition to the sub-tropical jet which is normally present. Such double-jet regimes have become more frequent in recent years due to high-latitude land warming, something which is partly attributable to anthropogenic greenhouse gas forcing^76^. Double jets favor waveguide formation and wave-resonance but the evidence for an increase in frequency or persistence of waveguides is limited^66,69^.

## Combined effects

The described dynamical mechanisms do not operate in isolation but instead interact with each other, with other teleconnections, and regionally with land-atmosphere feedbacks. The exact nature of these interactions is still speculative at this stage but positive feedbacks are certainly possible which would lead to tail risks.

Weakened storm tracks favor the buildup of hot and dry conditions over the continents. This can strengthen the land-ocean thermal contrast which, combined with the enhanced wave response to thermal forcing when the background flow is weak, could lead to amplified quasi-stationary waves. The projected weakening of storm tracks and westerlies can interact with soil moisture and snow cover changes, potentially regionally exacerbating zonal-mean circulation changes. Enhanced terrestrial heating in mid- to high-latitudes associated with reductions in snow cover can promote anomalous ridging in the upper troposphere, as suggested by both observational and modeling studies^53,97,98^. The emergence of such positive geopotential height anomalies in mid-to-high latitudes induces hot and dry air through subsidence and easterly wind anomalies of continental origin^60^. Likewise, drying soils in subtropical regions like the Mediterranean (a robust projection in future climate) can favor the formation of a heat low, i.e., low pressures at the surface due to upflow associated with intense surface heating^99^. A Mediterranean near-surface heat low brings easterly winds to central and western Europe, obstructing the normal westerlies^100^. Weakened or diverted westerlies can reduce rainfall leading to further soil drying. Due to such feedback mechanisms between soil moisture, snow cover changes, and continental-scale circulation, summer weather in western Europe and interior North America is likely to become more continental as seen in models^60,100,101^ and possibly also in observations^24,25,102^. The effectiveness of this mechanism depends on the delicate balance between land-atmosphere feedbacks and the strength (and thus its long-term weakening) of the storm tracks.

Recent studies indicate that the Atlantic Meridional Overturning Circulation (AMOC, i.e., the large-scale north-south transport in the Atlantic ocean) has seen an unprecedented slowdown in recent decades^103,104^, something which is projected for future warmer climates as well. This slowdown results in anomalously cold SSTs over the northern Atlantic which can trigger a quasi-stationary Rossby wave response favoring blocking high-pressure systems over western Europe^105^. So, just like AA, a slowdown of the AMOC leads to weakening westerlies in summer over the Atlantic sector, favoring persistent hot-dry extremes over Europe^106^. Recent observational studies indeed indicate that weather persistence in Europe and some other mid-latitude regions has increased in boreal summer^23–25,102^.

The Northern Hemisphere summer circulation is connected to the tropics primarily via two-way interactions with monsoon systems. Anomalously low snow cover over Eurasia during late winter to early-spring can increases the land-ocean temperature gradient due to snow-albedo and soil moisture effects which strengthens the Indian summer monsoon rainfall^107^. On sub-seasonal time scales, extreme monsoonal rainfall over eastern or western Himalayan foothills is often linked to southward intruding mid-latitude troughs, possibly part of the CGWT^108^. The monsoon is also a source of diabatic heating that can generate the CGWT^44^. Thus, wave trains originating from higher latitudes can modulate the intensity of the monsoon, and the monsoon’s strength in turn reinforces the downstream propagation of the wave train^45^. The projected increase in Indian summer monsoon rainfall is thus expected to strengthen the CGWT.

## Robust evidence and knowledge gaps

Several arguments support the AA influence on summer circulation as compared to winter. First, in the mid-latitudes, the equator-to-pole near-surface temperature gradient has seen a pronounced reduction in summer, but not in winter^26^. Second, late-spring to early-summer snow cover extent, which influences summer flow regimes, has dramatically declined^48,109^; and finally, summer circulation is less affected by tropical ENSO forcing, and thus potentially more sensitive to Arctic warming^17,38,44^.

Reviewing the literature, we conclude that there is robust evidence that AA causes a weakening of storm tracks in summer. The mechanism is straightforward: Cyclone genesis is directly related to the lower troposphere temperature gradient which weakens with AA. Further support comes from observed trends and historic and future climate modeling experiments. Still, multi-model attribution analyses are needed to quantify the exact role of past and future AA on the weakening of storm tracks.

There is still substantial uncertainty in what implications this weakening will have for summer weather conditions. Does it become more persistent and therefore more extreme? Theoretically, a weakened westerly flow leads to a small increase in the stationary wavenumber (i.e., a 5% decrease in the flow would lead to a 2.5% increase in stationary wavenumber^63^) but not necessarily to a more-stationary flow. Both observations and climate models suggest that a weaker westerly flow is associated with a more wavy flow pattern, especially in summer, but this does not necessarily imply a cause-effect relationship^60,61^ (see Box 3). A reduction in synoptic activity favors the buildup of hot-dry conditions and this can interact with regional land-atmosphere processes that can feedback on the continental-scale circulation (as outlined above). However, the relative role of these processes, and the strength of their interactions is largely unquantified. To assess future high-end risks, these non-linear interactions need to be disentangled and quantified.

There is robust evidence that summer time quasi-stationary waves lead to persistent and therefore extreme weather conditions. However, substantial uncertainty remains in how such waves will change under global warming including the role of AA therein. While physical mechanisms exist that could amplify quasi-stationary short waves as an indirect response to AA, their representation in climate models is biased^38^ and their relative importance compared to other drivers is poorly understood^46^. Some upward trends in quasi-stationary wave activity have been reported but confidence in these trends is generally low due to the use of different diagnostics leading to conflicting results (Box 3). Moreover, the relative importance of enhanced high-latitude warming, strengthened monsoons, and a weakening AMOC with associated mid-latitude SST anomalies, in shaping the mid-latitude summer circulation needs to be understood. All these drivers have a tendency to strengthen CGWTs but their interactions and regional effects are poorly known.

## Ways forward

Given the societal risks and large uncertainties, we argue for coordinated research efforts to address the knowledge gaps described above. To efficiently address this overarching theme, tighter collaboration between sub-disciplines within climate sciences is desirable, including scientists studying Arctic processes, monsoons, storm track dynamics, (sub) seasonal forecasts, and extreme weather.

Idealized atmosphere models are useful to study individual teleconnections and their drivers. However, a central challenge is to quantify the interactions between Arctic teleconnections and other teleconnections and regional processes, requiring state-of-the-art weather or climate models. Teleconnections are generally state-dependent (e.g., the Arctic’s influence might be pronounced only if the tropics are in a specific state) and non-stationary. While often the mean circulation response to a certain perturbation (e.g., removing sea-ice) is analyzed, it will be important to quantify the response in probabilistic terms: The change in frequency of certain high-impact circulation regimes such as amplified and persistent quasi-stationary waves. An increased frequency in such rare regimes will have little influence on the mean circulation but can have large societal impacts. To do so will require large ensembles and well-coordinated experiments with a range of different models. To disentangle the influence of different teleconnections, a so-called storyline approach can be insightful as well. This avoids quantifying probabilities associated with dynamical changes altogether and, instead, creates a set of physically plausible scenarios (i.e., storylines) of future changes^110,111^. For example, the change in European summer circulation could be described by combining a set of storylines that are based on the response to remote drivers from the Arctic, the tropics, AMOC, and regional soil-moisture changes. This way, the high-end risk (e.g., when Arctic and tropical teleconnections combine with soil-moisture feedbacks to push European summer weather towards much more persistent hot-dry conditions) can be identified and studied without assigning a specific probability to it^110^.

To understand and overcome model biases, e.g., in the representation of summer Rossby waves^112^ and ocean-atmosphere feedbacks in the presence of sea-ice^113^, novel machine learning approaches should be used to better integrate information from observations in climate models. In particular, causal discovery algorithms can identify causal pathways in the atmosphere and quantify their relative importance from observations alone^114^. They can thus be used to do process-based model validations and quantify how well models represent certain causal pathways in the atmosphere. This can give direct insight into the underlying physical reasons behind model bias and thus provides concrete targets for model improvements.

Finally, high-resolution paleo-climate records over the Holocene period can provide further insights into the circulation response to temperature gradient changes and put recent trends into a long-term perspective. Paleo proxies typically measure some form of biological activity and are therefore best suited to analyze the summer/growing season. The mid-Holocene provides a possible paleo analog with enhanced high-latitude warming and, interestingly, this period was also characterized by enhanced drought conditions in the mid-latitudes^115^. Likewise, tree-ring analyses suggest that enhanced jet stream waviness during summers in recent decades has been unprecedented over the post-1725 period^116^.

## Summary

Future impacts from extreme weather are likely to be most pronounced in summer, as most ecological activity and agricultural production takes place in this season^117^. Though the uncertainties are large, changes in atmosphere dynamics have the potential to cause rapid transitions at a regional scale leading to surprises for society. In summer synergistic effects between thermodynamic and dynamic drivers of extreme weather could act in the same direction to cause very-extreme extremes^17^. Recent summers have seen such anomalous weather (Box 1) and these events are not well understood. This presents risks for society and in particular for global food production, given that the major breadbasket regions are located in the mid-latitudes with many crop types vulnerable to heat extremes^118^.

The current literature provides robust evidence that AA influences mid-latitude summer circulation substantially by weakening the storm tracks. The uncertainties to do with other dynamical aspects and with how dynamical changes ultimately affect regional weather conditions are admittedly large. Nevertheless, we identified several possible feedback mechanisms for how storm track weakening can lead to persistent and therefore extreme weather in the mid-latitudes. Several studies suggest that Northern Hemisphere summer weather is indeed already becoming more persistent^23,24,66,119^.

In summary, this review shows that AA is likely to have substantial impacts on mid-latitude summer circulation. The societal impacts can be severe due to tail risks arising from radiatively forced mean summer warming combined with local and remote processes that favor more persistent summer weather. A coordinated research agenda focusing on summer circulation, its drivers and extremes is needed to resolve the key knowledge gaps.
